# Post-Adversities Recovery and Profitability: The Case of Italian Farmers

**DOI:** 10.3390/ijerph16173189

**Published:** 2019-09-01

**Authors:** Donatella Porrini, Giulio Fusco, Pier Paolo Miglietta

**Affiliations:** 1 Department of Economics and Management, University of Salento, 73100 Lecce, Italy; 2 Faculty of Science and Technology, Free University of Bolzano/Bozen, 39100 Bolzano/Bozen, Italy

**Keywords:** insurance, agriculture, Italy, risk management, adversities

## Abstract

Insurance represents one of the main instruments, together with other risk management mechanisms, to face the adverse effects produced by natural calamity that, despite their growing intensity and the enormous costs, are still perceived as “exceptional”. Risk management is an important part of farming, and it is a concern for those governments which aim at achieving their agricultural policy targets. In this context, crop insurance can also represent a financial mitigation tool for farmers to face climate change consequences. This study is focused on the Italian case analyzing the evolution of public support and its effect on risk management policy in agriculture. Our research, based on panel data regressions, provides two different levels of analysis. The first one evaluates how the reimbursed value issued by insurance companies in favor of agricultural firms, as recovery from natural adversities, affects farmers’ profitability. The second one evaluates how the reimbursed value is used in farm management. The results of the analysis demonstrating the significance of insurance variables and their positive effect on the profitability of the farms, represent a strong advance in the farm risk management field

## 1. Introduction

Forecasts by meteorologists and researchers of climatic changes provide that, apart from global warming, we must expect large quantities of downfalls and violent weather storms that would destroy agricultural cultures to a great extent. As a consequence, farmers and national governments need to specify measures that would protect agricultural cultures from threatening weather conditions and mitigate the loss of crop yields.

As a matter of fact, at a global level market penetration of agricultural insurances remains low, mainly due to under-estimated risks, and financial illiteracy, as well as limited supply [1]. This phenomenon of low demand is particularly widespread in low- and middle-income countries, despite the importance of the agricultural sector in many of these countries.

Elsewhere, especially in high-income countries, catastrophic risk management in agriculture is often reliant on public interventions, such as ex-post payments or price guarantees rather than explicit insurance tools [2]. In this case, low demand for insurance may be due to the availability of aid and financial assistance following a disaster. These responses, triggered by principles of solidarity and shared responsibility, contribute to underinsurance as they weaken the incentives to take ex-ante measures to reduce financial risk. Heavy reliance on government or private assistance is referred to as “charity hazard” [3]. A recent paper by Miglietta et al. [4] has empirically verified the existence of charity hazard in the Italian agricultural insurance system.

Generally, there is a tendency to replace government aid for natural disasters with a strengthened subsidized insurance system, but compared to national risk management policy in agriculture, it is challenging to explain the effects of such policies and the efficiency of public intervention. The main difficulties derive from the different type of effects connected to the policies [5]. In this sense, the attitude of governments towards payments in case of disasters is very important, favoring the adoption of traditional public or private instruments [6], or even the introduction of new tools [7].

Following the recent evidence of Capitanio and De Pin [8] and Zubor-Nemes et al. [9], our main research question regards the opportunity for the farmers to adopt insurance instruments obtaining a post-adversity recovery. The latter is a set of measures established by the insurance companies, including mainly a monetary reimbursement for damages, aimed at ensuring farm current activities, perdurability and ability to produce a profit. The present study contributes to extending the existing scientific literature on this topic, providing two different levels of empirical analysis. The first one addressed at evaluating how the reimbursed value issued by insurance companies, as a result of adversities, impacts the profitability of the farmers. The second one aimed at evaluating the effects of the reimbursed value on farm management.

## 2. Literature Review

Different studies stated that climatic changes affect the agricultural sector [10,11]. Crop growing is much more exposed to climatic conditions during the production processes [12,13] and, for several months, crops are under the influence of the agro-climatic conditions [14].

Other studies, showing a correlation among poverty, climatic changes and adversities’ effects on farms, underlined the need to introduce a model able to measure the consequent impacts and the necessary recovery systems [15,16].

Part of the scientific literature focused on the analysis of climate change impacts and adaptation strategies among different countries. Mutekwa [17] appraised the knowledge of the farmers on the risks produced by the climatic changes in Zimbabwe. Similar studies were conducted in Scotland, England, Brazil and Tanzania [18,19,20].

Risk management in agriculture has become a matter of greater interest in the last decades [21,22,23], and different studies tried to deepen farmers’ beliefs and concerns about climate change. Glenk and Fischer [24] provided a study in Scotland to see how the citizens perceived the public policies concerning climate change. Arbuckle et al. [25] analyzed farmers’ knowledge about the damages produced by climatic changes.

An integrated bio-physical and bio-economic farm model has been applied to croplands in Austria with the aim to evaluate the impacts of climate change and mitigation and adaptation policy scenarios on farm production, as well as on the environment [26]. Addressing specifically water management purposes, Steidl et al. [27] analyzed the impact of climate change and increased irrigation area on future hydrologic and agro-economic conditions for a representative basin in Northeastern Germany, while Nunes et al. [28] evaluated the impact of climate and associated socio-economic changes on water availability in a southern region of Portugal, already characterized by a dry Mediterranean climate and a drought stress.

Other studies have discussed which instrument is the best for risk management in agriculture. In his study Tangermann [29] analyzed the possible sources of risks and potential management strategies deepening the effects of EU Common Agricultural Policy. More recently Cortignani and Dono [30] evaluated the potential impact of the reform of the first pillar of CAP in two different climate scenarios, simulating the possible adaptation of various farm types in an agricultural area of Southern Italy to changes, given the available technological options and current market conditions.

Since agriculture is considered one of the most sensitive sectors to climate changes, many studies focused on the development of financial recovery tools [31,32,33].

A large part of the literature about agricultural insurance has asserted that without subsidies no farmers would insure themselves, but the use of a targeted system, with costs charged to farmers, can lead to a reduction in problems of moral hazard and political failure [34,35]. Agricultural insurance has stimulated a strong interest among politicians and scientists proved by numerous studies aimed at assessing the value and the effect of agricultural insurance in a different part of the world [36,37,38,39,40,41]. In order to evaluate the efficiency of the insurance system in the agricultural sector, Lorant and Farkas [42] compared insurance systems in the different OECD countries; in this perspective many studies have analyzed the impact of insurance as a risk management tool and its social and ecological consequences [43,44,45,46].

A combination of policy and climate uncertainty could effectively impact agricultural production, land use and farm income [47]. In recent years, in fact, many studies discussed the relation between agricultural insurance and profitability for farms [34,48,49]. In the wake of this literature emerge the research questions of this study, addressed to assessing the effects of reimbursements on the profitability of the farmers and on agricultural management.

## 3. Background: The Italian Case

In Italy, national policy to support risk management in agriculture has undergone major changes. In 1970, when the government provided agricultural insurance, policy was based essentially on the establishment and financing of a national solidarity fund (FSN) to compensate the damage suffered by farmers following natural disasters, while the support for the payment of insurance premiums was secondary.

The system based on ex-post compensation for damage worked well enough in periods when the incidence of adverse events was sporadic, but when the frequency of adverse weather events increased, this form of intervention showed its limits.

For this reason, the government decided to modify policy intervention in the management of risks in agriculture in Italy, particularly for the management of damage caused by natural disasters.

Changes in legislation started in 2002, continuing in the following years, until reaching its peak in 2004 with a Legislative Decree 102/2004, approved in line with the European regulatory framework. The subsidy is granted up to 80 percent of the premiums for insurance contracts that provide compensation if the damage reaches 20 percent of production in disadvantaged areas and 30 percent in other areas. If insurance contracts also cover other losses, due to adverse weather conditions not assimilated to natural disasters or plant diseases, the subsidy is reduced by up to 50 percent of the insurance premium.

Since2005, the public subsidy is granted exclusively for insurance contracts, which provide coverage of the farm’s overall production within the same municipality.

The insurance subscription is voluntary and can take place collectively or individually. The defense consortia, as well as agricultural cooperatives and their consortia, may decide to resort to collective insurance.

One of the main reasons justifying public support for insurance in agriculture—which takes the form of both the contribution to the payment of policies underwritten by farmers, and the benefits to companies in terms of reinsurance of the risks assumed—stems from the alleged failure of the private market to provide such insurance.

Besides reducing market failures, public intervention would also increase overall efficiency, allowing the market to offer a service for which there would be a demand, as it has the potential to address information asymmetry caused by systemic risk, adverse selection and moral hazard, as stressed by Cafiero et al. [50].

In assessing insurance systems, it is necessary to take into consideration aspects related to systemic damage and adverse selection. Systemic damage occurs when a lot of insured persons are simultaneously damaged by an adverse event affecting a geographical area. It is a problem considered typical in agricultural insurance, due to adverse weather events, which, by striking relatively large areas, cause simultaneous damage to a large number of farms. The systemic nature of the damages means that it is possible to distribute risks among the group of insured persons in the absence of spatial correlation of adverse events.

**Figure 1:**
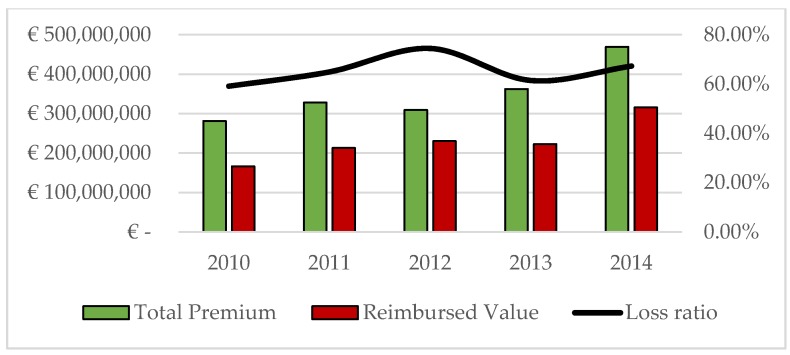
The evolution of the insurance system in Italy 2010–2014. Source: Personal elaboration on SICURAGRO data [51].

Trying to assess the impact of the systemic nature of damages in agricultural insurance in Italy, Figure 1 shows the ratio between compensated claims and premiums paid at a national level. The loss ratio at a national level is always less than one unit and ranged between a maximum of 75% in 2012 and a minimum of 59% in 2010.

The data available from the database SICURAGRO do not provide information on the profits made by the insurance companies operating in agriculture. An indication, although general, is offered by the loss ratio. Since the loss ratio index fluctuated between 0.59 and 0.75, insurance companies never received higher compensation than the premiums collected, presumably making good profits over the entire period examined.

**Figure 2:**
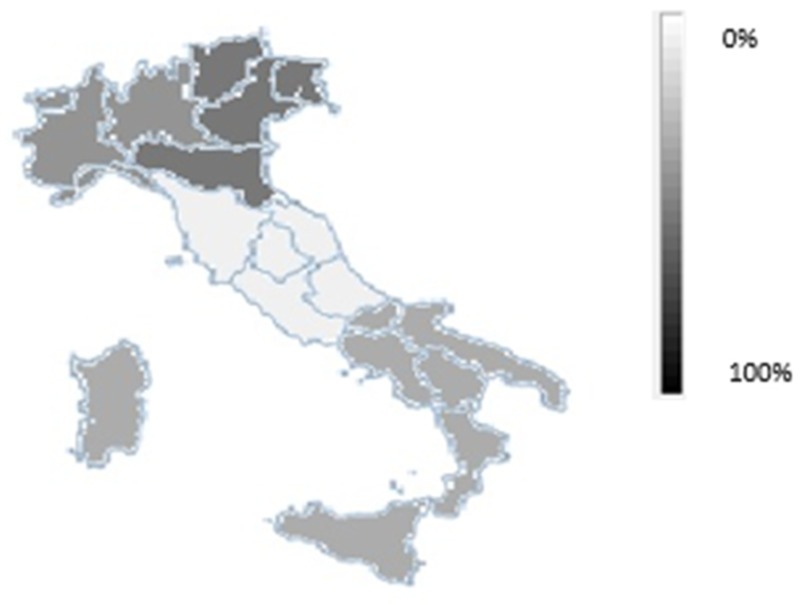
Geographical distribution of agricultural insurance. Source: Personal elaboration on SICURAGRO data [51].

Figure 2 describes the spatial distribution of agricultural insurance in Italy and shows that almost all insurance certificates issued are located in the North, with 52% in the North-East and 27% in the North-West; whereas in the South and in Central Italy, they are not very widespread, accounting for just over 20%. This disparity across Regions also reflects the different value of the agricultural production.

## 4. Materials and Methods

The main source of data for our empirical research is the Farm Accountancy Data Network (FADN), provided by the Directorate-General Agricultural and Rural Development of the European Commission, where there are all economic and accounting data for the Agricultural Holdings [52]. The latter are single units, both technically and economically, operating under single management and which undertake agricultural activities within the economic territory of the European Union.

Particularly, the data about phytosanitary products, total cultivated surface, agricultural production, labor force, total intermediate consumption, gross farm income and depreciation, related to agricultural holdings for the 2010–2014 period and aggregated for each Italian Region have been extracted from FADN (Table 1) and selected among those used by the established literature [53].

The *Phytosanitary products* variable refers to the quantities of fertilizers, and soil improvers (excluding those used for forests) purchased on average by the agricultural holdings for each Region and expressed in Euro (€).

The *Agricultural production* variable represents instead the total output of crops and crop products, corresponding to the sum of sales, farm use, farmhouse consumption and closing valuation minus opening valuation, achieved on average by the agricultural holdings of each Region, expressed in Euro (€).

The *Labor force* in agriculture is measured by the annual working units, i.e., fulltime person equivalents, (expressed in thousands) employed on average in the agricultural holdings for each Italian Region.

The *Total cultivated surface* measures the average hectares (ha) of the total utilized agricultural area (UAA) destined to cultivation of all types of crops, excluding areas used for mushrooms, land rented for less than one year, woodland and the other farm areas. It is made up of land in owner occupation, rented land, land in sharecropping AND includes agricultural land temporarily not under cultivation for agricultural reasons or as a result of being withdrawn from production as part of agricultural policy measures.

The *Total intermediate consumption* variable represents the difference between gross output and net output, obtained on average by the agricultural holdings of each Italian Region. It includes total specific costs (including inputs produced on the holding) and overheads arising from production in the accounting year.

The variable *Gross Farm Income* represents the average value registered by agricultural holdings for each Region, obtained by the difference between the total output crops and crop production (Sales + farm use + farmhouse consumption) and the total intermediate consumption summed to the balance current subsidies and taxes.

The *Depreciation* variable represents the total amount of depreciation of capital assets over the accounting year, registered on average by the agricultural holdings of each Region. It concerns plantations of permanent crops, farm buildings and fixed equipment, land improvements, machinery and equipment and forest plantations and is determined on the basis of the replacement value.

Data about the *Reimbursed Values* for the same time window, aggregated for each Italian Regions, have been acquired from the Database on Agricultural Hazards (SICURAGRO) [51]. This Risk Database in Agriculture was established by ISMEA (Istituto di Servizi per il Mercato Agricolo Alimentare) by a Decree of the Italian Ministry of Food and Forestry Policies of 18 July 2003 and aims at supporting public intervention for the agricultural risk management and at providing informative elements for shareholders, even for the purpose of risk prevention.

The *Reimbursed value* variable represents the amount of money recovered on average by the farmers, after the adversities, thanks to the insurance contract. This value is expressed in thousands of Euro (1000 €).

Finally, *Position* is a dummy variable assuming value 0 and 1 for each Region, where 0 indicates a geographical location in Southern Italy or the Islands (Sicily and Sardinia) and 1 indicates a geographical location in Northern Italy.

Previous studies analyzed the effects of agro-climatic variables on agricultural production, using dynamic fixed effects models [54], but also on insurance aspects, using pooled OLS regression [41].

This study addressed two empirical research questions. The first one is aimed at evaluating how the reimbursement, obtained by agricultural firms as recovery from natural adversities, affects the farmers’ profitability. The second one aims at investigating how the reimbursement is employed in farm management. A panel data analysis has been conducted for the time period 2010-2014, through the latest version of the statistical software GRETL, frequently used in econometrics to analyze two-dimensional panel data.

The models proposed in this study are estimated for 19 Italian Regions with the soleexclusion of Valle d’Aosta, due to the lack of data. Specifically, we considered the following independent variables: *Phytosanitary Product*, *Total Cultivated Surface*, *Agricultural Production*, *Labor force* and *Reimbursed value*.

In order to investigate the impacts of an insurance variable on agricultural holding profitability, we used the *Total intermediate consumption*, the *Gross farm Income* and the *Depreciation* as dependent variables, capturing different aspects of farm profitability, in line with other studies [55,56]. The most widely used econometric structure to investigate the effects of risk management through insurance variables on profitability is as follows:

(1) $$\begin{array}{l} Lny_{i,t}=\alpha_{1}+\beta_{1}\;LnReimbursed\;Value_{i,t}+\beta_{2}Phyto_{i,t}+\beta_{3}\;LnAgrProd_{i,t}\\ \;+\beta_{4}LnTotal\;Surface_{i,t}+\beta_{5}LnAgr\;Lab_{i,\;t}+\beta_{6}\;Position_{i,}+\varepsilon_{1,\;t}, \end{array}$$

where the parameter of interest *y* is an economic variable (in our case Total Intermediate Consumption, Gross Farm Income and Depreciation), which captures how the economic performance of the farmers in each Italian Region acts in relation to the independent variables i.e., *Ln Reimbursed Value*, *Ln Phyto*, *Ln Agr Prod*, *Ln Total Surface*, *Ln Agr Lab* and *Position*, while $\varepsilon_{1,t}$ is the error term.

We have conducted three regressions to provide the robustness of empirical results.

(2) $$\begin{array}{l} LnGross\;Farm\;Income_{i,t}\\ \;=\alpha_{1}+\beta_{1}\;LnReimbursed\;Value_{i,t}+\beta_{2}Phyto_{i,t}+\beta_{3}\;LnAgrProd_{i,t}\\ \;+\beta_{4}LnTotal\;Surface_{i,t}+\beta_{5}Ln\;Labor\;force_{i,t}+\beta_{6}\;Position_{i,}+\varepsilon_{1,t} \end{array}$$

(3) $$\begin{array}{l} LnTotal\;Intermediate\;Consumption_{i,t}\\ \;=\alpha_{1}+\beta_{1}\;LnReimbursed\;Value_{i,t}+\beta_{2}Phyto_{i,t}+\beta_{3}\;LnAgrProd_{i,t}\\ \;+\beta_{4}LnTotal\;Surface_{i,t}+\beta_{5}Ln\;Labor\;force_{i,t}+\beta_{6}\;Position_{i,}+\varepsilon_{1,t} \end{array}$$

(4) $$\begin{array}{l} LnDepreciation_{i,t}\\ \;=\alpha_{1}+\beta_{1}\;LnReimbursed\;Value_{i,\;t}+\beta_{2}Phyto_{i,t}+\beta_{3}\;LnAgrProd_{i,t}\\ \;+\beta_{4}LnTotal\;Surface_{i,t}+\beta_{5}Ln\;Labor\;force_{i,t}+\beta_{6}\;Position_{i,}+\varepsilon_{1,t} \end{array}$$

In Table 2 we show the summary statistics of the sample:

## 5. Results and Discussion

Before proceeding with the analysis, normality tests were conducted on the sample data. Results highlighted significantly that the data follows a Gaussian distribution. For this reason, parametric procedures can be used.

In order to evaluate the relationship between the variables considered, their linear correlation was calculated (Table 3). The correlation analysis indicates how the insurance independent variable *Reimbursed Value* is strongly correlated to the dependent variables *Gross Farm Income*, *Total Intermediate Consumption* and *Depreciation*. The positive relationship among variables derives from the importance of risk management in the agricultural holdings [57,58].

The columns labelled (1), (2), and (3), included in Table 4, report the results of three-separate panel regressions with fixed effect. The values in the Table are the coefficients, standard errors (in parentheses), their *p*-values, and summary statistics, as indicated by the description in each row.

The first column of labeled regression results (1) in Table 4 considers a linear relationship between the dependent variable *Ln Gross Farm Income* and the independent variables. The second column of labeled regression results (2) in Table 4 considers a linear relationship between the dependent variable *Ln Intermediate Total consumption* and the independent variables. Finally, regression (3) describes the relationship between the dependent variable *Ln Depreciation*, and our independent variables.

In the first regression, we can see how the only significant variables are *Ln Reimbursed Value*, *Agr Prod* and *Tot Surface*. In particular, the relationship between our dependent variable and the insurance variable is significant (*p*-value < 0.001) and negative, in particular, a variation of 1% of Reimbursed Value originates consequent negative variation around of 0.3% in the *Gross Farm Income* variable. The first regression analysis shows a close positive relationship between *Gross Farm Income* and *Agr Prod*, the same effect can be observed focusing on *Tot Surface*.

Focusing on the summary statistics of regression, it is possible to notice that the *adjusted R^2^* assumes a value equal to 0.821 quantifying the extent to which the explanatory variables explain the variation in the dependent variable.

The second model, described in column (2) of Table 4, presents different results compared to the first model; the reason for this difference is that, in this case, we have considered a net economic variable *Ln Total Intermediate consumption*. The result of the second regression demonstrates how the adoption of insurance instruments is very important for the Agricultural Holding. We have a positive significant (*p*-value < 0.001) relationship between the independent insurance variable (*Ln Reimbursed*) and the dependent variable, and for the variables *Agr Prod and Tot Surface* we have a similar result of the first model. As a demonstration that our independent variables better explains the variation in the dependent variable, the value of *adjusted R^2^*, in this case, is equal to 0.901.

The third variable considered is *Depreciation*. The result of the analysis shows that the insurance variables relation to the dependent variable is not significant. In the third regression, only the *Position* variable has a significant relationship, which indicates that the Agricultural Holdings located in the North have a higher depreciation than the Agricultural Holdings located in the South. In this case, the *adjusted R^2^* assumes a value equal to 0.558.

By the result of the first regression it is possible to affirm that the adoption of insurance instruments is important for the Agricultural Holdings, the negative significant relationship indicates that the insurance instruments cover the losses, but the reimbursed value is not enough to cover the total amount of losses.

In the second regression, the significant and positive relationship between the reimbursed value and the economic value (*Total Intermediate consumption*) gives us important information about the impact of insurance instruments. In particular, this relationship indicates the importance of insurance instruments on the profitability of the Agricultural Holdings because the reimbursed value is used by farmers to guarantee the total monetary value of goods and services consumed or used as inputs in production.

The results of the third regression demonstrate that Italian farmers do not use the reimbursed value to reduce the depreciation of capital assets. This circumstance is proved by the non-significance of each coefficient in the third regression and by the R^2^. The latter, in fact, is lower than the other two regression analyses, demonstrating that depreciation is not well explained by the independent variables considered in this study.

In this sense, the absence of insurance impacts cash flow for several problems such as the need for farmers to buy new machinery or to replace plants [59].

As emerged from our empirical research, in which we have demonstrated the effect the insurance variable on the profitability of agricultural holdings, it is very important to improve risk management, especially in case of adversities, spreading the insurance tools as much as possible.

## 6. Conclusions

The analysis shows that the main weakness in the Italian system is represented by the territorial differences, in terms of insurance policy diffusion, between the Northern and Southern-Insular Regions. In the latter, the spread of insurance tools is very low, with a value of around 25%. This prevents the public budget from being able to offset ad hoc compensatory interventions in the case of disastrous events, due to the low transferability of damages related to the insurance market.

The reasons for this situation can be different and numerous. On the one hand, this may be due to the inadequacy of insurance contracts with respect to the demand for risk management by farmers in the Center and the South of Italy. On the other hand, the farmers in this area could have a lower propensity than Northern farmers to adopt instruments due to the transfer of risk to third parties. The farmers’ perspectives in using insurance instruments are connected to their experiences, i.e. previous receipt of indemnity or significance yield losses in the past [60].

A further disincentive for taking up insurance is the uncertainty about the availability of public funds to support insurance premium payments, which was a major brake on the evolution of the sector. This situation made it impossible to introduce multi-year contracts, which could have reduced the problems deriving from information asymmetry between farmers, insurers and government.

The results in Section 5 confirm how insurance represents one of the most important drivers to achieve profitability for firms that could be affected by natural disasters.

Specifically, in line with Park et al. [61], our results denote how the global prize connected to the insurance system represents one of the most important drivers to preserve the capability of firms to make a profit. In fact, the negative effects of natural disasters could increase the possibility for firms to achieve negative profit, due to the sub-sequentially increase of the managerial risks [62].

The efficiency of the system can be improved through the increase in the number of insured parties and a wider coverage offered by the contracts that would reduce the costs of managing policies and compensation for damages. Also, with the introduction of insurance contracts on area yields, there could be a strong increase in the overall efficiency of the system, by reducing the problems induced by the information asymmetry and the costs for damages. These types of contracts, which are very common in the USA, are struggling to take off in Italy, due to the lack of information necessary for their implementation.

A further goal of public intervention should also be to increase the self-insurance potential of farmers against less serious risks at the farm level, by inviting and supporting farmers to use multiple private tools and improve their active protection (e.g., anti-terrorist networks, irrigation against drought). At the same time, future scientific research should propose incentives aiming at exploring the opportunities offered by innovative instruments, such as indexed insurance on climate parameters. Institutional intervention should try to reduce the informational asymmetries in the market by encouraging the adoption and the diffusion of such an insurance instrument that is still used below its potential.

## Figures and Tables

**Table 1:** Sources of data.

Source of Data	Code	Data Acquired	Time Period	Unit of Analysis
FADN [52]	SE275	Total intermediate consumption (€)	From 2010 to 2014	Italian Regions
	SE420	Gross Farm Income (€)		
	SE360	Depreciation (€)		
	SE025	Total cultivated surface (ha)		
	SE010	Labor force (1000 annual working units)		
	SE135	Agricultural production (€)		
	SE295	Phytosanitary product (€)		
SICURAGRO [51]	-	Reimbursed Value (1000 €)		
ISTAT	-	Position		

**Table 2:** Summary statistics.

Variables	Mean	Standard Deviation	Minimum	Maximum
Total cultivated surface (ha)	17.60	8.479	3.19	44.79
Agricultural production (€)	42,257.28	19,272.03	18,080.00	109,339.00
Total intermediate consumption (€)	26,808.65	17,876.46	6237.00	92,625.00
Phytosanitary product (€)	2408.14	1054.75	900.00	6720.00
Labor force (1000 annual working units)	1.38	0.34	1.01	2.95
Reimbursed Value (1000 €)	87,302.74	229,570.35	0.00	1,566,012.92
Position	0.63	0.485	0.00	1.00
Gross Farm Income (€)	39,009.26	17,137.16	18,286.00	106,522.00
Depreciation (€)	7300.22	3033.50	2829.00	14,326.00

**Table 3:** Correlation analysis.

Variable	Reimbursed Value	Agr Surface	Phyto	Agr Prod	Labor Force	Gross Farmer Income	Total Intermediate Consumption	Depreciation	Position
Reimbursed value	1	0.255	0.066	0.626	0.755	0.054	0.324	0.198	0.205
Agr Surface		1	0.191	0.250	0.237	0.210	0.184	0.133	0.159
Phyto			1	0.504	0.113	0.792	0.747	0.480	0.429
Agr prod				1	0.807	0.601	0.742	0.543	0.568
Labor force					1	0.332	0.512	0.408	0.358
Gross Farmer Income						1	0.889	0.547	0.467
Total intermediate consumption							1	0.608	0.503
Depreciation								1	0.579
Position									1

**Table 4:** Regression results.

	*Ln Gross Farm Income*	*Ln Total Intermediate Consumption*	*Ln Depreciation*
*Ln Reimbursed Value*	−0.0339896 *** (0.0105740)	0.0697444 *** (0.0116548)	0.0266593 (0.0274813)
*Ln Phyto*	0.155033 (0.14762)	0.349052 ** (0.133130)	0.148146 (0.210690)
*Ln AgrProd*	0.664713 *** (0.163423)	0.655473 *** (0.142480)	−0.0456099 (0.338077)
*Ln Labor force*	0.0765265 (0.23467)	0.339970 * (0.168154)	0.587118 (0.377879)
*Ln TotSurf*	0.339613 *** (2.00138)	0.322562 *** (0.0781898)	0.175566 (0.126330)
*Position*	0.0606357 (0.0447147)	0.106262 * (0.0581844)	0.397054 *** 0.0803349)
*Constant*	1.62297 * (0.803597)	−1.33879 * (0.737767)	6.97767 *** (2.10362)
**Summary Statistics**			
SER	0.187119	0.21127	0.323536
Adjusted *R*^2^	0.821	0.901	0.558
N. observation	95	95	95

Standard errors are given in parentheses under coefficients. Individual coefficients are statistically significant at the 10% (*), 5% (**) or 1% (***) level.
